# 

**Published:** 1899-01

**Authors:** 


### LIST OF CONTRIBUTORS TO VOLUME XX.

Allen, Dr. Frederick W.
Baker, George T., D.D.S.
Bishop, Dr. Burton F.
Boenning, Henry C., M.D.
Brooks, George, D.D.S.
Brubaker, Albert P., M.D., D.D.S.
Bryan, L. C., D.D.S.
Cardwell, Dr. Harold.
Catching, B. H., D.D.S.
Darby, Edwin T., M.D., D.D.S.
Dawbarn, Robert H. M., D.D.S. M.D.
Delabarre, Frank A., D.D.S. M.D.
Dickinson, E. B, D.D.S.
Engs, John S., D.D.S.
Gray, George R., D.D.S., D.M.D.
Greenbaum, Leo, M.D., D.D.S.
Hayter, Mark, D.D.S.
Heyke, Dr. John E.
Hodgkin, Dr. J. B.
Hubbard, Dwight L., M.D.
Jameson, G. L. S., D.D.S.
Kirk, Edward C., D.D.S.
McCutcheon, W. H., D.D.S.
Meriam, Horatio C., D.D.S.
Parmele, George L., M.D., D.M.D.
Palmer, S. B., M.D.S.
Pond, Virgil C., B.P., D.M.D.
Rogers, F. T., M.D.
Rollins, William, D.D.S.
Royce, Waldo E., D.D.S.
Schamberg, Jay F., A.B., M.D.,
Schamberg, Morris I., M.D. D.D.S.
Smith, D. D., M.D., D.D.S.
Smith, F. Milton, D.D.S.
Smith, Dr. Theo. V.
Southwick, George R., M.D.
Stevenson, Frederick A., D.M.D.
Strang, C. W., D.D.S.
Talbot, Eugene S., M.D., D.D.S.
Trueman, William H., D.D.S.
Truman, James, D.D.S.
White, Dr. Harry F.
Whitney, J. M., D.D.S.
Willoughby, Edward F., M.D.
Younger, W. J., M.D.

